# For Chronic Bronchitis

**Published:** 1901-04

**Authors:** 


FOR CHRONIC BRONCHITIS.

BUTLER (*Medical Standard*) says that in a majority of cases balsam, turpentine and tar derivatives are the most efficient expectorants, being especially indicated in relaxed conditions of the mucous membranes, with excessive secretion, in combination as follows:

| | | | |
|---|---|---|---|
| ℞ | Oil of turpentine . . . . . . . . . . . . . . | 1.33 | mxx. |
| | Liquid tar . . . . . . . . . . . . . . . . . . | 1.33 | mxx. |
| | Oil of eucalyptus . . . . . . . . . . . . . . | 3.33 | ml. |
| | Balsam of tolu . . . . . . . . . . . . . . | 6.00 | ʒiss. |
| | Benzosal . . . . . . . . . . . . . . . . . . . | 16.00 | ʒiv. |

Mix. Dispense in sixty (60) capsules. Dose—One capsule four or five times a day.

